# Supplementary material for: Polyketide synthase gene clusters in the ericoid mycorrhizal fungus Oidiodendron maius: genome-wide prediction, expression profiling, and functional characterization of knockout mutants
Source: Front Plant Sci. 2026 Sep 16;17:1924430. doi: 10.3389/fpls.2026.1924430 (PMC13623903; doi:10.3389/fpls.2026.1924430)
Supplement: Supplementary file 1 [file SupplementaryFile1.zip › Supplementary Materials/Supplementary Folder 1.zip!/Oidma1_AssemblyScaffolds_Repeatmasked/knownclusterblast/cluster3/jgi.p_Oidma1_107176_mibig_hits.html]

| MIBiG Protein | Description | MIBiG Cluster | MiBiG Product | % ID | % Coverage | BLAST Score | E-value |
| --- | --- | --- | --- | --- | --- | --- | --- |
| Solyc12g006470.1 |  | BGC0001319\_c2 | alpha-Tomatine\_biosynthetic\_gene\_cluster. | 30 | 94.8936170213 | 201 | 2e-51 |
| ABY66011.1 | aminotransferase | BGC0001008 | Maduropeptin\_biosynthetic\_gene\_cluster | 32 | 77.6595744681 | 165 | 2.1e-40 |
| ADY76679.1 | aminotransferase | BGC0000950 | Napsamycin\_biosynthetic\_gene\_cluster | 30 | 82.1276595745 | 161 | 1.8e-39 |
| ADN26241.1 | PLP-dependent\_aminotransferase | BGC0000951 | Pacidamycin\_biosynthetic\_gene\_cluster | 30 | 75.5319148936 | 155 | 1.3e-37 |
| ABV97170.1 | aminotransferase\_class-III | BGC0000137 | Rifamycin\_biosynthetic\_gene\_cluster | 29 | 93.829787234 | 151 | 2.4e-36 |
| CAB39702.1 | probable\_aminotransferase | BGC0000663 | Hopene\_biosynthetic\_gene\_cluster | 29 | 91.7021276596 | 149 | 7e-36 |
| ABB69077.1 | putative\_3-acetyloctanal\_aminotransferase | BGC0000260 | Prodigiosin\_biosynthetic\_gene\_cluster | 30 | 79.3617021277 | 149 | 7e-36 |
| ACH85568.1 | CetH | BGC0000283 | Cetoniacytone\_A\_biosynthetic\_gene\_cluster | 30 | 77.6595744681 | 141 | 3.3e-33 |
| EFE73302.1 | N2-acetyl-L-ornithine:2-oxoglutarate\_aminotransferase\_ArgD | BGC0000431 | Stenothricin\_biosynthetic\_gene\_cluster | 29 | 93.4042553191 | 140 | 5.6e-33 |
| AAY93435.2 | 2,4-diaminobutyrate\_4-transaminase | BGC0000413 | Pyoverdine\_biosynthetic\_gene\_cluster | 26 | 98.7234042553 | 112 | 1.6e-24 |
| AEU11006.1 | NpnB | BGC0001029 | Nostophycin\_biosynthetic\_gene\_cluster | 27 | 67.8723404255 | 73 | 6.4e-13 |
